# Exploratory multivariate analysis of the effect of fatty fish consumption and medicinal use on heart rate and heart rate variability data

**DOI:** 10.3389/fpsyg.2015.00135

**Published:** 2015-02-17

**Authors:** Bjørn Grung, Anita L. Hansen, Mari Berg, Maria P. Møen-Knudseth, Gina Olson, David Thornton, Lisbeth Dahl, Julian F. Thayer

**Affiliations:** ^1^Department of Chemistry, University of BergenBergen, Norway; ^2^Department of Psychosocial Science, University of BergenBergen, Norway; ^3^Centre for Research and Education in Forensic Psychiatry, Haukeland University HospitalBergen, Norway; ^4^Sand Ridge Secure Treatment CentreMauston, WI USA; ^5^National Institute of Nutrition and Seafood ResearchBergen, Norway; ^6^Department of Psychology, The Ohio State UniversityColumbus, OH, USA

**Keywords:** heart rate, heart rate variability, fatty fish, medicine, multivariate data analysis

## Abstract

The overall aim of the present study was to explore the relationship between medicinal use and fatty fish consumption on heart rate variability (HRV) and heart rate (HR) in a group of forensic inpatients on a variety of medications. A total of 49 forensic inpatients, randomly assigned to a fish group (*n* = 27) or a control group (*n* = 22) were included in the present study. Before and by the end of the food intervention period HR and HRV were measured during an experimental test procedure. An additional aim of this paper is to show how multivariate data analysis can highlight differences and similarities between the groups, thus being a valuable addition to traditional statistical hypothesis testing. The results indicate that fish consumption may have a positive effect on both HR and HRV regardless of medication, but that the influence of medication is strong enough to mask the true effect of fish consumption. Without correcting for medication, the fish group and control group become indistinguishable (*p* = 0.0794, Cohen’s *d* = 0.60). The effect of medication is demonstrated by establishing a multivariate regression model that estimates HR and HRV in a recovery phase based on HR and HRV data recorded during psychological tests. The model performance is excellent for HR data, but yields poor results for HRV when employed on participants undergoing the more severe medical treatments. This indicates that the HRV behavior of this group is very different from that of the participants on no or lower level of medication. When focusing on the participants on a constant medication regime, a substantial improvement in HRV and HR for the fish group compared to the control group is indicated by a principal component analysis and *t*-tests (*p* = 0.00029, Cohen’s *d* = 2.72). In a group of psychiatric inpatients characterized by severe mental health problems consuming different kinds of medication, the fish diet improved HR and HRV, indices of both emotional regulation and physical health.

## INTRODUCTION

Psychiatric inpatients are often on a variety of medications, and it is known that the life expectancy for this group is lower than the expectancy for the general population (Nome and Holsten, 2012). Studies have also shown that people suffering from psychiatric disorders often have deficiencies in key nutrients such as omega-3 fatty acids and vitamin D (Partonen, 1998; Perica and Delas, 2011). Diet has shown to have a profound effect on both physical health, e.g., cardiovascular diseases (CVDs; Van Horn et al., 2008), and the mental health (Timonen et al., 2004). Self-reporting survey studies and nutrient supplement studies dominate this research field, due to the difficulties in conducting a well-controlled diet intervention study. Because of the low life expectancy in psychiatric inpatients on a variety of medications more knowledge about effective health care interventions is needed. Due to the fact that fatty fish is an important source of essential fatty acids and vitamin D, nutrients important for both physical and mental health (Lansdowne and Provost, 1998; Christensen et al., 2001a,b; Ross et al., 2007; Dobnig et al., 2008; Giovannucci et al., 2008), results from controlled fish intervention studies may have important implications.

Although many studies report on the effects a steady diet of fatty fish may have on the physical and mental health, the number of studies examining the underlying mechanisms are much rarer. Heart rate variability (HRV), which is an objective and important index of both physical and mental health (Thayer and Lane, 2007), has been mentioned as one such mechanism. HRV is a measure of beat-to-beat changes in the heart rate (HR), and is a measure of the interplay of the sympathetic and parasympathetic branches of the autonomic nervous system. In itself HRV is a predictor of sudden death in patients with coronary heart disease (Kleiger et al., 2005), which makes the relationship between fatty fish consumption and HRV interesting to investigate. A reduction in HRV is regarded as an indicator of future fatal or near-fatal cardiac arrhythmia (Huikuri et al., 2009), and it is thus an important measure of an individual’s physical health. Thayer and Lane developed a model of neurovisceral integration (Thayer and Lane, 2000, 2007, 2009) that connects prefrontal cortex activity with that of the heart. This theory provides a physiological explanation as to why HRV not only predicts physical, but also emotion- and self-regulation. Several later studies (Hansen et al., 2003, 2004, 2009) have underpinned the model by associating HRV with executive functions, the underlying mechanism involved in emotion- and self-regulation (Shimamura, 2000; Thayer and Lane, 2000). Mozaffarian et al. (2008) found that changes in various HRV parameters were related to increased fatty fish intake. This was done by controlling for factors such as age, gender, ethnic background, education, smoking, alcohol intake, body mass index, diabetes mellitus, prevalent coronary heart disease, β-blocker use, physical activity, and intakes of beef, pork, fried fish and total calories. However, it is not well established whether the effect of fish consumption on HRV is direct or indirect (Mozaffarian et al., 2008). It has been speculated whether serotonin may be involved in the effect (Hansen et al., 2010) due to the fact that both vitamin D and omega-3, which are found in fatty fish, are important for the regulation of serotonin and serotonin is further important for the regulation of HRV (Stumpf and Privette, 1989; Hibbeln et al., 2006).

The use of HRV as an indicator of mental and physical health status is not problem free. A recent paper by Quintana and Heathers (Quintana and Heathers, 2014) highlights the impact of respiration on HRV. Several external factors, such as stress, age, physical shape and sickness affect the HRV, and may thus disturb the results. CVD may of course disturb the beat pattern of the heart, and persons suffering from depression tend to have lower HRV than the normal population (Kemp et al., 2010). There is a strong link between depression and CVDs, as a substantial number of CVD patients suffer from depression (Carney et al., 1987; Gonzalez et al., 1996), and persons diagnosed with depression run a higher risk of certain CVDs (Anda et al., 1993; Barefoot et al., 1996; Pratt et al., 1996; Penninx et al., 2001). Another major influence on HRV is medication. Blood pressure medications (Bonaduce et al., 1997; Pinar et al., 1998), cholesterol medication (Riahi et al., 2002; Welzig et al., 2003; Gomes et al., 2010), β (Cook et al., 1991; Mølgaard et al., 1993; Niemelä et al., 1994; Sandrone et al., 1994; Ebbehøj et al., 2002) - and α- blockers and ACE inhibitors (Guedon-Moreau et al., 1997; Shehab et al., 2008) are all examples of heart medications with either known or suspected effects on HR and HRV. Among antidepressants, the most commonly used are selective serotonin reuptake inhibitors (SSRI). Serotonin itself plays a role in the HRV regulation, and an effect of SSRI on HRV can be expected. Still, various studies come to different conclusions as to whether SSRI influences HRV. In their recent review and meta-analysis, Kemp et al. (2010), concluded that some antidepressants, although successful in easing the symptoms of depression, did not improve HRV significantly. As patients with depression have a higher risk for developing CVD, a treatment that remedies the psychological symptoms, but leaves the physiological ones untreated, may imply a lower longevity for this group. Some studies (Balogh et al., 1993; Khaykin et al., 1998) demonstrate a positive effect, whereas others (Licht et al., 2010) show a negative effect on HRV. For other types of antidepressants the situation is similar, and no consensus seems to have been arrived at (van Zyl et al., 2008) on their possible effect on HRV.

Recently it was demonstrated that fatty fish consumption improved sleep quality and resting HRV (Hansen et al., 2014a). However, in that study only the effect of high frequency (HF) HRV was investigated and other HRV parameters such as low frequency (LF) and the LF/HF ratio were not investigated. Thus, a combined effect of different HRV parameters was not investigated. Moreover, no attempts were made to investigate the effect of various medications used by the participants on their psychophysiological reactivity to different conditions, such as an experimental mild-stress procedure consisting of a baseline period (resting), a variety of different cognitive tasks taxing executive functioning and a recovery (resting) period. Executive functioning tasks require focused attention over prolonged periods of time in order to be performed accurately. Carrying out these tasks can be a stressful experience. Both increased HR and decreased HRV have been found during the performance of such tasks (Porges and Raskin, 1969). Since HRV is associated with mortality, and psychiatric inpatients on a variety of medications are known to have a lower life expectancy than the general population (Nome and Holsten, 2012), investigation of HRV reactivity to different conditions while controlling for medication may yield important information of general daily autonomic activity patterns.

A major aim of this study was to generate hypotheses concerning the effects of a fatty fish diet on the HRV and HR reactivity in a group of psychiatric patients characterized by severe mental health problems consuming many different kinds of medications. Here, we focus on heart medication and anti-depressants administered on a regular basis. In order to generate new hypotheses the investigation is carried out in an exploratory way using multivariate data analysis (Principal Component Analysis and Partial Least Squares), with focus on easily interpretable plots. This technique represents a valuable addition to the battery of statistical tests normally carried out. Because of the different findings in the literature, it was also investigated whether any effect on HRV and HR could be detected prior to intervention and assigned to the usage of anti-depressants. Finally, it was investigated whether the data indicated that users of such medication experienced a different effect of the fatty fish intake compared to the participants who did not use such medication.

## MATERIALS AND METHODS

### PARTICIPANTS

The present study was part of a larger research project concerning the effects of fatty fish consumption (Hansen et al., 2014a), where 95 male forensic inpatients characterized by severe emotional disturbances (e.g., antisocial personality disorders, borderline personality disorders, generalized anxiety and major depression) had been randomly assigned into a Fish group and a Control group. In the current study a subset of 49 participants were suitable for an investigation of whether the intake of medications had an impact on the effect of the fish consumption. Details about the study progress are reported in Hansen et al. (2014a). The number of subjects in the fish group was 27. The number of subjects in the control group was 22. During the test period five subjects withdrew from the fish group, and six from the control group. Details on then randomization procedure can be found in Hansen et al. (2014b).

### APPARATUS

Physiological activity was measured by recording HR and HRV using the Actiheart System (Cambridge Neurotechnology Ltd; Brage et al., 2005), a compact lightweight device that records HR and variability of R-R inter-beat intervals. The Actiheart clips onto a single ECG electrode (M-00-S/50 Blue Sensor) with a short ECG lead to another electrode that detects the ECG signal. The Actiheart was placed on the upper chest.

The Actiheart provides interbeat intervals with a resolution of 1 ms, which are used to calculate HR and HRV. HR was defined as the average HR in beats per minute for an analysis epoch of 1 min. In the frequency domain, HRV was measured as absolute high frequency power (HF; 0.15–0.4 Hz), absolute low frequency power (LF; 0.04–0.15 Hz) and their ratio (LF/HF). HF and LF were derived by fast Fourier transform of the spectrum. The HF component is known to reflect primarily parasympathetic influences. However, concerning the LF there is some controversy. It has been argued that LF reflects both sympathetic and parasympathetic activity; others argue that LF reflects only sympathetic activity (Malliani et al., 1991; Appelhans and Luecken, 2006; Xhyheri et al., 2012). In the time domain, HRV was measured as the root mean of the squared successive differences (RMSSD) of the inter-beat interval. The RMSSD reflects parasympathetic activity. The data was log transformed prior to analysis (Malik and Task Force of the European Society of Cardiology the North American Society of Pacing Electrophysiology, 1996). Artifacts were manually cleaned by visual inspection using the Actiheart program.

### PROCEDURE

The study protocol and all experimental procedures were approved by the Ethics Committee at the facility in Mauston, WI, USA, and were in compliance with the Helsinki declaration for research ethics. Participants were recruited by both written and oral information about the study. Thus, the patients were invited to participate in a research project concerning nutrition and mental health. The participants were informed that the purpose of the study was to investigate if nutrition (fatty fish or an alternative meal like chicken, pork, or beef) would have any effects on mental health. They were also informed that they would be randomly assigned into two groups; one group that would eat *fatty fish* (portion size 150–300 g) three times a week and one group that would eat *meat* (e.g., chicken, pork, beef) meals three times a week for a period for 6 months (September–February). For information about the fish used in the intervention study, see Hansen et al. (2014a). The participants signed an informed consent form, and they were informed about their rights to withdraw from the study at any time for any reason without penalty. Thus, the study design was fully disclosed to all participants, and all participants provided informed consent.

Prior to (July) and toward the end (February) of the intervention period the participants went through a test procedure. Since the present study is part of a larger project concerning fatty fish consumption (Hansen et al., 2014a) these procedures involved collections of various kinds of data such as fasting blood sample, sleep data, self-report questionnaires as well as an experimental test procedure measuring psychophysiological activity during exposure to different executive function tasks. In order to obtain a broad measure of reactivity, psychophysiological activity was registered for 5 min of baseline (rest condition), during exposure to the different executive function tasks (i.e., Iowa Gambling Task, Tower of Hanoi, and N-back tasks; 0–3 back), and during 5 min of recovery (rest condition). The length of executive tasks varies from task to task, and person to person. Thus, the HRV was registered for a total period of 50–60 min. During these periods of time, one measurement epoch lasts for 1 min. This means that there are five measurements for baseline and recovery (five epochs of 1 min during a 5 min interval), and a varying number of measurements for the tasks. All participants were exposed to exactly the same experimental procedure before (pre) and after (post) the food intervention period. All participants were tested individually.

### MEDICINAL USE

Copies of all medical records, from 1 month prior to 1 month after intervention, were available for a subset of participants. The various drugs administered were classified according to the Anatomic Therapeutic Chemical Classification System, which is controlled by the World Health Organization. For each participant, daily dosages of each pharmaceutical used continuously for at least 1 week prior to the pre-test period were registered. This was done to avoid inclusion of drugs to which the participants had not yet properly responded. The same procedure was carried out for the post-test. For the forthcoming data analysis, only categories C (cardiovascular drugs) and N06A (anti-depressants) were used actively in the analysis.

Some participants were excluded from the data analysis. Participation in the project was voluntary, and three participants withdrew for various reasons during the intervention period. Participants released from the facility or transferred to a different facility during the study were included in the analysis of the pre-test, but are of course not present in the post-data. Participants with partially missing journals were excluded from further analysis. For some participants, a substantial amount of missing data was observed in the HR and HRV data. Participants with more than 20% missing data were excluded from further analysis.

### THE DATA TABLES

A data matrix was constructed from the pre-test data. It contained HR and HRV data for 49 participants. For each participant there were 40 variables recorded. These were the HR and the four HRV measures (LF, HF, LF/HF, and RMSSD) recorded during the eight stages of the experiment: baseline, the six psychological tests and recovery. A similar matrix was created from the post-intervention test data. To highlight the effect of the intervention on the HR and HRV, the pre-test data was subtracted from the post-test data, thus yielding a matrix reflecting the change in HR and HRV measures. The resulting table is hereafter referred to as the change data. The creation of this matrix necessitated a full set of data from both before and after the intervention, and thus only 38 participants are included in this matrix. Five of the 11 participants lost to follow up belonged to the fish group, and six belonged to the control group. Data was logarithmically (base 10) transformed prior to analysis. Due to the variables being measured using different units of measurements, autoscaling was carried out whenever HR and HRV data was used in the same analysis. For analysis using only HR or HRV data, mean centering of the variables was carried out.

### THE DATA ANALYTICAL TOOL

The data analytical tool used in the investigation here may be unfamiliar to many readers. Principal Component Analysis (PCA; Joliffe, 2002) and Partial Least Squares (Manne, 1987) belong to a group of methods which may be referred to as latent variable techniques. They are methods frequently employed in chemometrics and related fields. The strength of these methods comes from their ability to deal with collinear data structures, in which the variables exhibit correlations. Another advantage of these methods is the easily interpreted plots that result from such analysis, and a presentation of this is to be found in the Results section.

While a more detailed mathematical explanation can be found in, e.g., Manne (1987) and Joliffe (2002), a brief explanation is given here. Latent variable methods take a set of correlated, measured variables (like the HR and HRV data in this study) and create a new, smaller set of orthogonal latent variables. The latent variables are linear combinations of the measured variables, and may be constructed using different criteria. In PCA, the latent variables (called principal components) are constructed using the maximum variance criterion. This means that the set of principal components are the best lower-dimensional approximation of the data, and that they represent the major sources of variation in the data. This makes PCA well suited for exploratory analysis.

Just like any object (participant in our study) has a value for each measured variable, all objects have a *score* for each principal component. Similarly, the contribution of each measured variable to the latent variable is expressed as the variable’s *loading*. Bivariate scatter plots of the first two score or loading vectors give the best two-dimensional presentation of the major correlation structures in the data, and reveal similarities and differences among the objects and variables in a data matrix. This soft modeling approach can be regarded as a hypothesis generator, since it visualizes relationships in the data that may otherwise go unnoticed. Latent variables can also be used to confirm or verify preconceived assumptions and hypotheses, since it is possible to carry out statistical tests on the latent variables. In the present work, the emphasis has been on data exploration, and less on quantification and hypothesis testing.

For the creation of regression models, other latent variable techniques are preferred. Rather than focusing on the variance in the predictors (the independent variables), PLS creates latent variables focusing on the covariance between the predictors and one or several response variables (the dependent variables). Because of the orthogonalization inherent in PLS, this regression technique does not suffer from the problem of collinear data that, e.g., multiple linear regression does.

The multivariate analyses were carried out using Sirius 9.0 from Pattern Recognition Systems AS, Bergen, Norway (www.prs.no).

## RESULTS

**Table 1** displays summary statistics for the HR and HRV data at pre- and post-test. For the executive functioning tasks (EF tasks) the average HRV and HR values for all cognitive tasks (Iowa Gambling Task, Tower of Hanoi, and N-back tasks; 0–3 back) are reported.

**Table 1:** Mean values (*M*) and SD for the heart rate (HR), the root mean square of successive differences (RMSSD), low frequency power (LF), high frequency power (HF), and the LF/HF ratio for both groups during pre- and post-test.

	Fish group			Control group		
	*M*	SD	*n*	*M*	SD	*n*
**Pre-test**
Baseline HR	72.96	13.15	27	72.81	13.13	22
Baseline RMSSD	1.44	0.28	27	1.45	0.27	22
Baseline LF	3.02	0.40	27	2.90	0.40	22
Baseline HF	2.22	0.59	27	2.29	0.49	22
Baseline LF/HF	0.82	0.33	27	0.64	0.26	22
EF tasks HR	71.48	13.06	27	70.12	11.82	22
EF tasks RMSSD	7.23	0.34	26	7.05	3.87	22
EF tasks LF	2.82	0.59	26	2.86	0.37	22
EF tasks HF	2.14	0.75	26	2.36	0.46	22
EF tasks LF/HF	0.69	0.27	26	0.53	0.22	22
Recovery HR	70.47	12.08	26	69.71	12.14	22
Recovery RMSSD	1.62	0.31	27	1.63	0.27	22
Recovery LF	3.30	0.43	27	3.32	0.39	22
Recovery HF	2.61	0.61	27	2.69	0.46	22
Recovery LF/HF	0.79	0.41	27	0.72	0.28	22
**Post-test**
Baseline HR	71.08	12.50	22	76.11	9.87	16
Baseline RMSSD	1.56	0.28	22	1.47	0.32	16
Baseline LF	3.11	0.40	22	2.99	0.59	16
Baseline HF	2.39	0.54	22	2.36	0.54	16
Baseline LF/HF	0.78	0.40	22	0.68	0.17	16
EF tasks HR	70.37	11.33	22	73.96	11.08	16
EF tasks RMSSD	1.54	0.36	22	1.52	0.26	16
EF tasks LF	2.92	0.47	22	2.81	0.49	16
EF tasks HF	2.37	0.70	22	2.38	0.49	16
EF tasks LF/HF	0.57	0.30	22	0.47	0.28	16
Recovery HR	69.75	9.98	22	75.17	10.81	16
Recovery RMSSD	1.63	0.30	22	1.49	0.23	16
Recovery LF	3.26	0.38	22	2.97	0.44	16
Recovery HF	2.61	0.62	22	2.44	0.40	16
Recovery LF/HF	0.73	0.33	22	0.60	0.27	16

*The HRV data are log-transformed. The number of participants in each group is in the column *n*.*

### PRE-TEST

**FIGURE 1:**
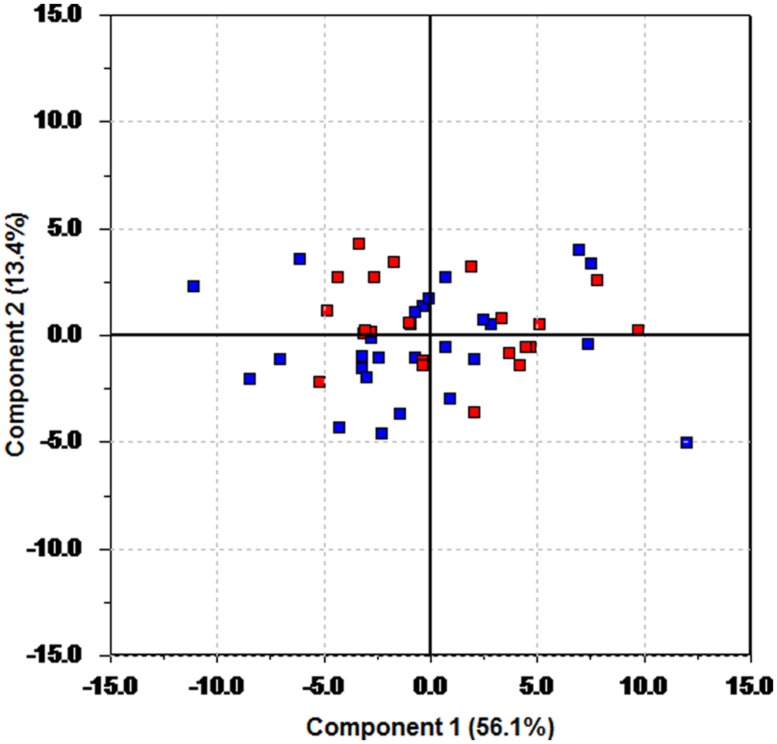
**Score plot of the first two principal components of the pre-test data.** Blue color is used for the fish group, and red for the control group.

Plots of data are often useful conveyors of information. The data matrix from the pre-test period contains 40 measurements for each of the 49 participants. A graphical display of the information content in such a large data matrix may seem difficult to obtain. One alternative is to plot each variable separately (thus creating 40 plots), or to produce all possible combinations of bivariate plots of one variable against another. This creates 780 plots, which of course is impossible to carry out. Through PCA a two-dimensional projection capturing most of the variation in the data can be obtained, resulting in a bivariate plot to be used for interpretation. For this particular data set, a two component (two latent variables) PCA model captures nearly 70% of the variance in the data. A bivariate plot of these two latent variables is shown in **Figure 1**, and it is referred to as a score plot. Since the axes in **Figure 1** are latent variables, information from all 40 measured variables is used to create this plot showing information on all 49 participants. This multivariate nature of the axes makes the plot an excellent tool for detecting relationships among the individual participants. In **Figure 1**, members of the fish group are plotted in blue, while red has been used for the control group. This information has only been used to color code the plots, and is not included in the data material.

There is no discernable trace of any grouping of the samples; the future fish and control group members are spread evenly throughout the plot. This indicates that the randomization strategy has been successful, as there is no visible group separation prior to intervention. A *t*-test on the scores on the first principal component shows that there is no significant difference in the mean value of the score values of the fish group compared to the control group (*p* = 0.38, effect size Cohen’s *d* = 0.25).

**FIGURE 2:**
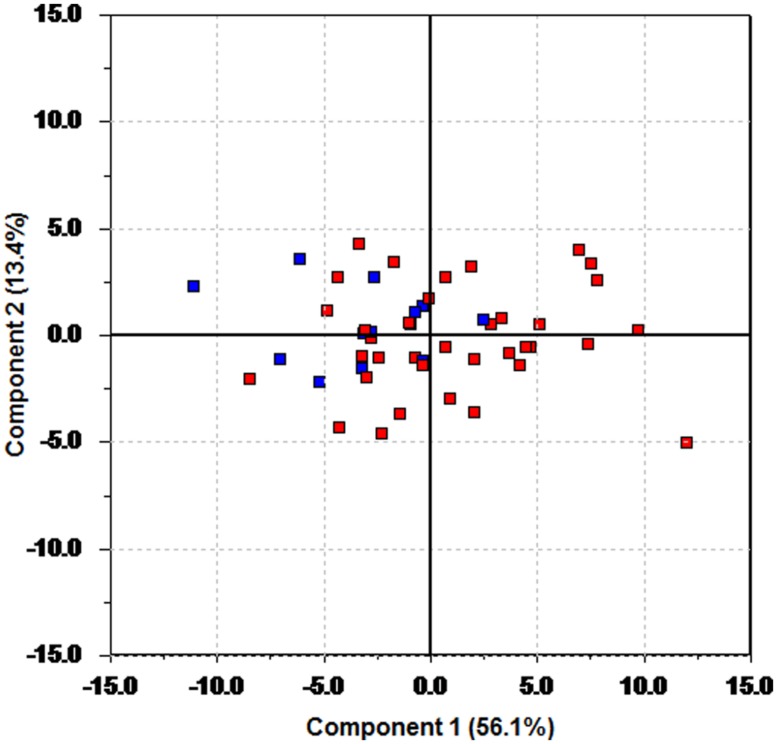
**Score plot of the first two principal components of the pre-test data.** Blue color is used for participants using anti-depressants, and red for those without such usage.

By changing the color coding (but not the underlying model) clustering can be revealed. **Figure 2** is the result of changing the color coding so that patients on anti-depressants are plotted in blue and those not using anti-depressants in red. The data analyzed and the resulting model is the same as in **Figure 1**, only the color coding has changed. **Figure 2** shows that patients using anti-depressants differ from the other patients by having a lower score on the first principal component. The findings from the PCA are confirmed by a *t*-test on the mean values of the scores on the first principal component. This results in a clear identification of a difference between the groups (*p* = 0.0037, effect size Cohen’s *d* = 1.01).

**FIGURE 3:**
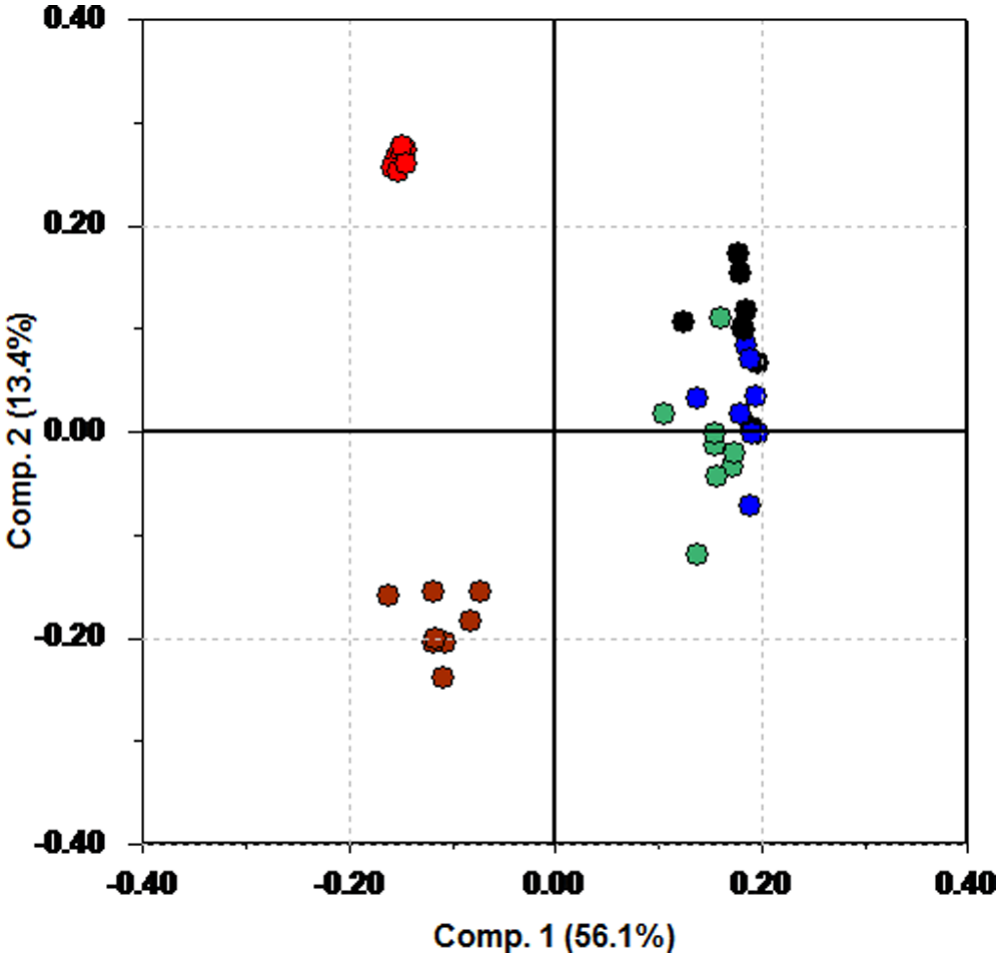
**Loading plot from PCA of the pre-test data.** Red is HR, blue is RMSSD, black is HF, green is LF, and brown is used for LF/HF.

While the score plot shows clustering, the corresponding loading plot is necessary to understand why groupings occur. **Figure 3** shows the loading plot. Again, color coding has been used. HR variables are plotted in red, and blue has been used for the RMSSD variables. The HF variables are in black, and green has been used for LF. The LF/HF ratio is plotted in brown.

**Figure 3** shows that participants with a low score on the first principal component (the patients using anti-depressants) have a tendency to have a higher HR (red) and LF/HF (brown) compared to the rest of the patients.

**FIGURE 4:**
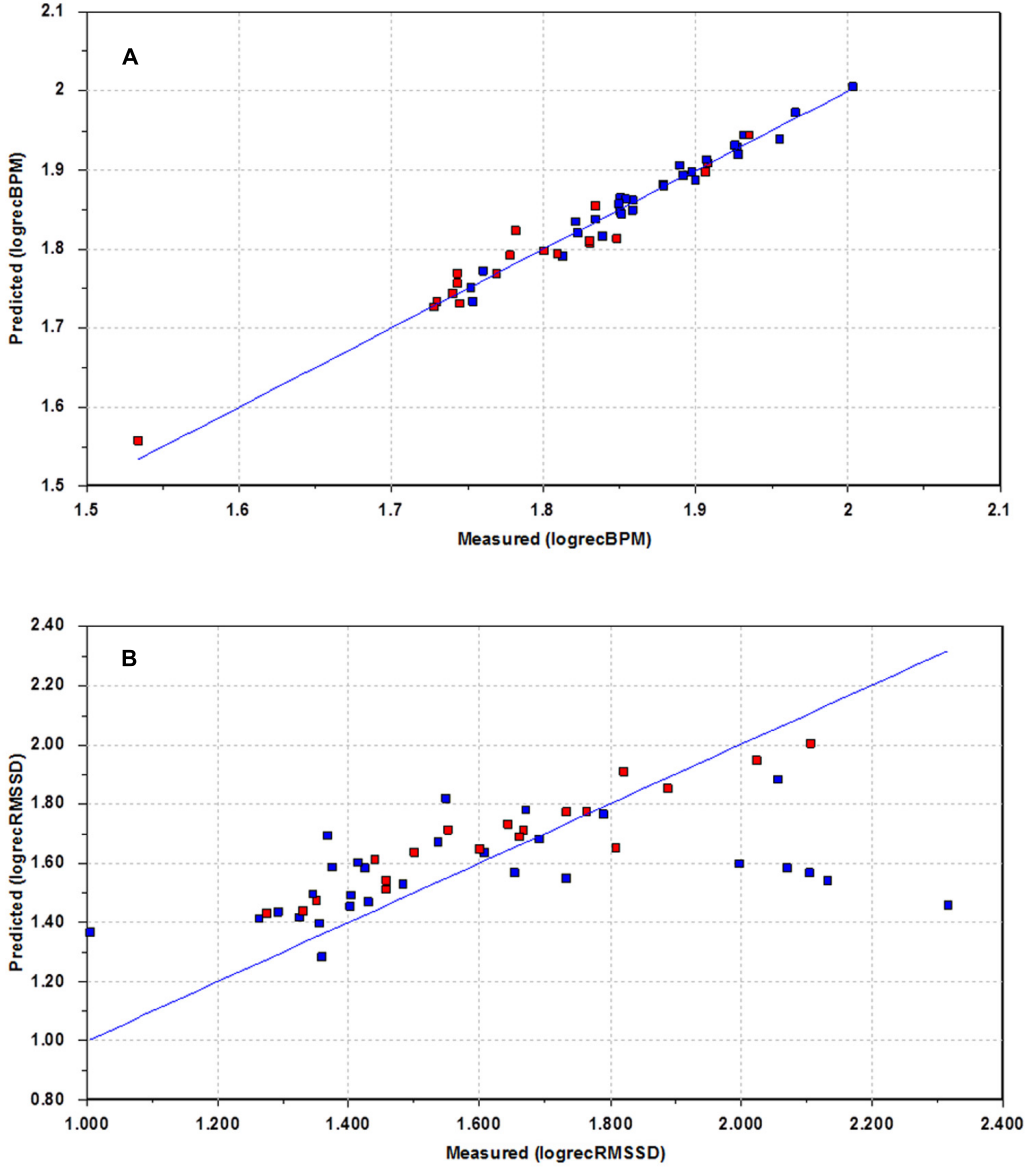
**Plots of estimated recovery versus measured recovery.** Blue – uses heart medication and/or anti-depressants. Red – does not use drugs. **(A)** Recovery HR. **(B)** Recovery RMSSD.

The recovery phase is an indicator on how the body adjusts to the normal situation after a period of mild stress. It was thought to be of interest to see if the patients on anti-depressants responded differently during recovery. This was done by creating PLS regression models that modeled recovery behavior as a function of behavior during baseline and the test period. Separate models were created for HR and each of the individual HRV measures. In **Figure 4A**, a plot of modeled recovery versus actual recovery for HR is shown. Again, blue color is used to denote participants using anti-depressants. The blue line represents perfect match between modeled and actual response. **Figure 4A** shows a good correspondence between measured and estimated HR for the whole group. The HR behavior during recovery is possible to estimate for all test participants, regardless of medication use.

This situation changes when looking at some of the HRV measures. **Figure 4B** illustrates the situation for RMSSD. For several participants the model performance is poor. For five of the participants (to the right), the actual RMSSD is far higher than expected based on the model. There is also one participant (to the left) with a measured RMSSD far lower than expected according to the model. This interesting result shows that this group of patients, all using anti-depressants, undergoes a different recovery phase with regards to RMSSD, compared to the other patients. A similar behavior is experienced when studying LF, but not for HF, or LF/HF.

### THE CHANGE DATA

The most important data table investigated in this work was created by subtracting the pre-test data from the post-test data. This was done to focus on each participant’s change in HR and HRV during the intervention. The score plot from PCA on this data set (not shown), shows little sign of discrimination between the fish and control group. A *t*-test on the scores confirms this (*p* = 0.0794, Cohen’s *d* = 0.60).

**FIGURE 5:**
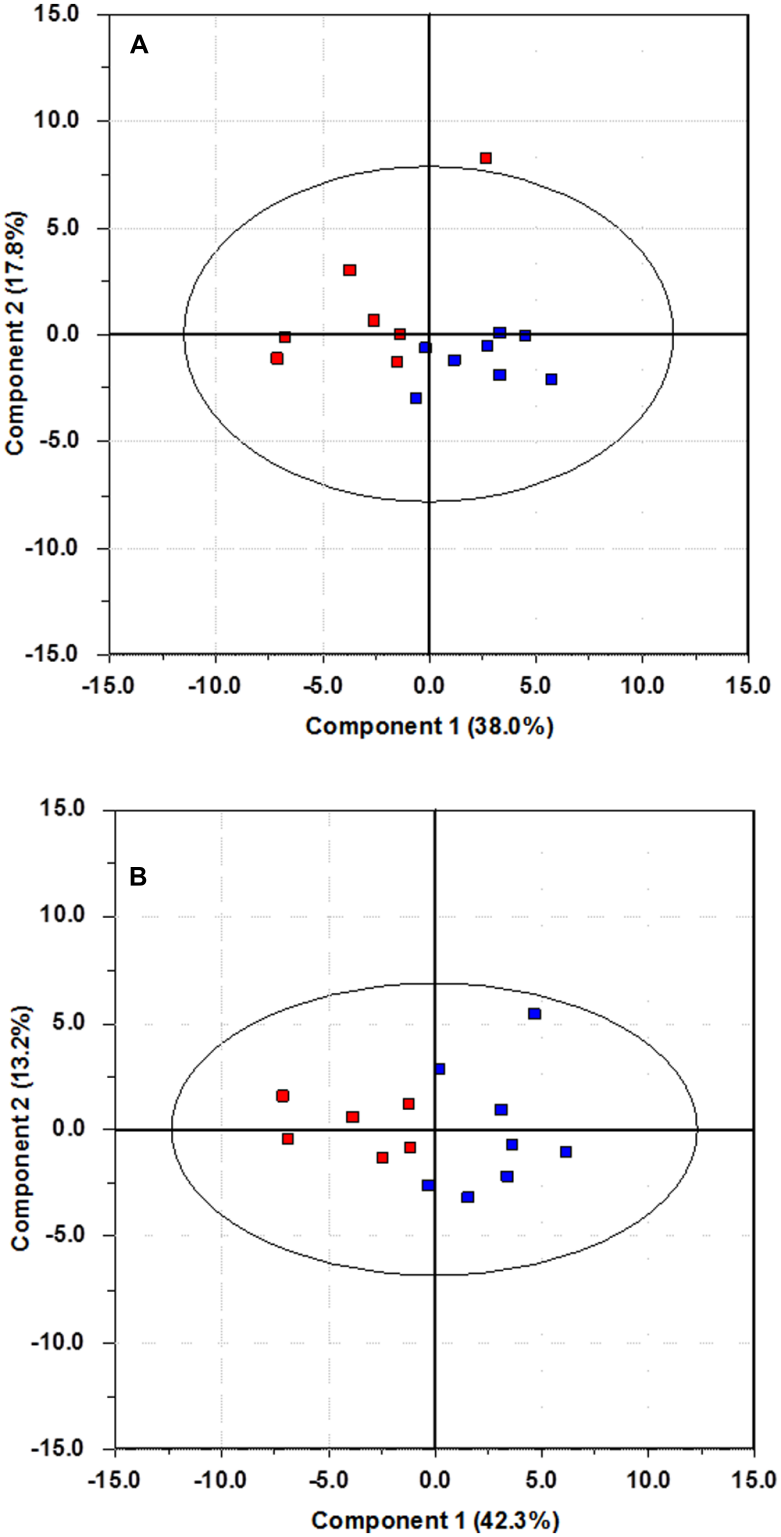
**Score plot of the first two principal components of the change data for the participants using neither heart medication nor anti-depressants.** Blue color is used for the fish group, and red for the control group. **(A)** Outlier included. **(B)** Outlier excluded.

However, clear separation between the fish and control group can be achieved by controlling for medication. In **Figure 5**, two score plots from PCA on a subset of participants free from heart medication and anti-depressants are shown. Again, blue represents intervention and red control. **Figure 5A** shows the results from a PCA on this subset of participants. The ellipses in the plot are Hotelling’s T^2^ limits (Jackson, 1991), which can be regarded as a multivariate generalization of a *t*-test. An outlier is present in the upper right quadrant of the plot. Removal of this outlier and recalculation of the model yields the score plot in **Figure 5B**. Separation is clearly visible, and a *t*-test on the mean value of the scores of the first principal component (*p* = 0.00029, Cohen’s *d* = 2.72) show that the two groups are different, and that the fish group has undergone a change in HR and HRV different to the intervention group.

**FIGURE 6:**
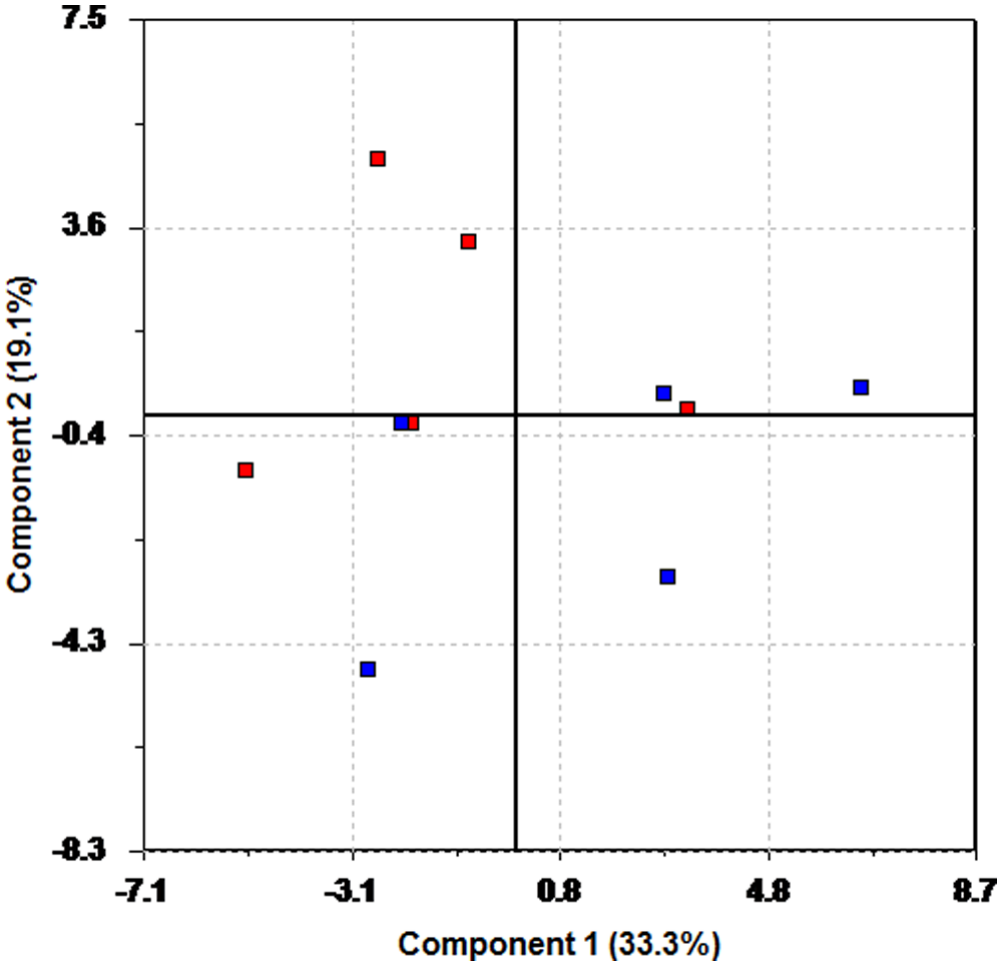
**Score plot of the first two principal components of the change data for the participants in the fish group having either a constant drug regime (blue) or receiving no medication (red)**.

A closer look at the status of subjects in the fish group is presented in **Figure 6**. This analysis was performed on the patients whose medication was constant throughout the intervention. Red is used to indicate the participants on constant medication, while blue is used for the ones who did not receive any medication. With the small sample size here, one should be careful with drawing strong conclusions. However, separation of the groups is evident from the score plot, as the patients not using medication is positioned more in the lower right part of the plot. This indicates a difference in response to the intervention. Still, the small sample size makes this observation less certain than the others presented in this work. We have therefore not carried out any additional statistical tests, but the plot generates the interesting hypothesis that the participants on medication respond differently to the fish diet.

**FIGURE 7:**
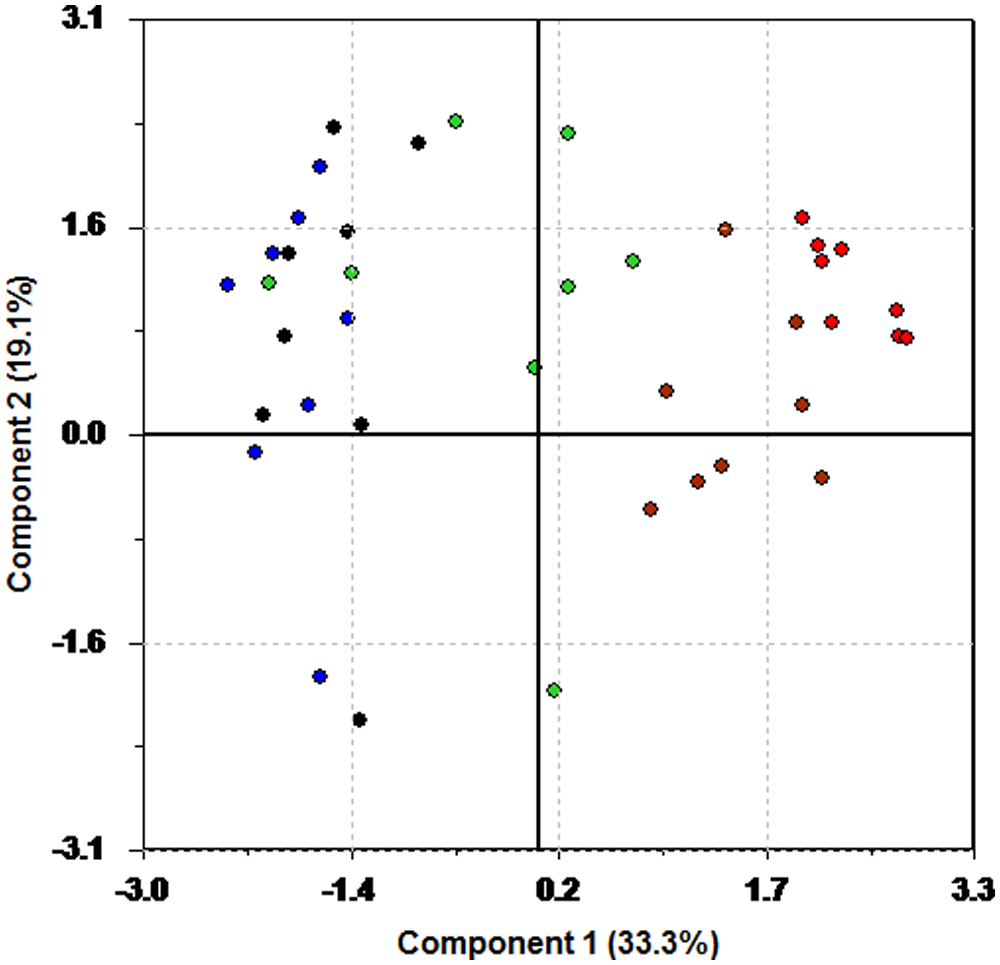
**Loading plot of the change data using only the participants in the fish group having a constant drug regime or receiving no medication.** Red: HR. Blue: RMSSD. Green: LF. Black: HF. Brown: LF/HF.

The loading plot in **Figure 7** illuminates why there is a separation. The same color coding is used in this loading plot as in **Figure 3**. HR variables are plotted in red, and blue has been used for the RMSSD variables. The HF variables are in black, and green has been used for LF. The LF/HF ratio is plotted in brown.

In the upper left region we find some of the LF, HF, and RMSSD measures, whereas larger change in HR and LF/HF measures seem to be associated with the participants not on medication.

## DISCUSSION

The use of latent variables as a data analytical tool is probably new to many readers. We do not suggest that these methods should replace traditional statistical tests, but rather complement them. Latent variables, in conjunction with statistical tests, showed a successful randomization procedure, as seen in **Figure 1**. A priori knowledge of group membership can still be used effectively by color coding the participants and variables accordingly to highlight the information contained in the variance pattern of the data. A PCA on the change data was initially unable to separate the fish group from the control group (*p* = 0.0794, Cohen’s *d* = 0.60). This is due to the criterion used to calculate the principal components. The maximum variance criterion forces the principal components to pick up the major variation sources, at the expense of leaving minor, systematic changes unaccounted for. The effect of medication was strong enough to hide the effect of the intervention. Only by controlling for medicinal use a separation of the two groups became visible in the score plots (*p* = 0.00029, Cohen’s *d* = 2.72). Although there of course are huge individual HR and HRV variations due to other external factors, the intervention group in general scores higher on the first principal component. This demonstrates that whether or not a participant is on the fish diet is a major source of variation in the data. A similar plot (not shown here) appears if one investigates only the subset of participants, whose medication remained constant throughout the intervention period, again indicating that the introduction of fatty fish to the diet is a major source of variation once medication is controlled for.

The use of quantitative regression modeling using latent variables has been shown for parts of the recovery data. The poor performance of the model for RMSSD prediction for some of the participants indicates that the recovery phase is different for some of the participants on heavy medications. For the majority of participants the model performance is good, so an interesting question is to ask why the model fails for some participants. A closer look at the medical records reveals that the participants in question are using a variety of heart medications and anti-depressants; up to eight different drugs at the same time. Thus, participants with a higher level of medication respond differently during the recovery phase to the normal population. Five of the six participants for which the prediction fails were using statins, a cholesterol lowering drug which previously has been shown to improve HRV (Riahi et al., 2002). Four participants were using fibrates, another cholesterol lowering drug which has been reported to improve HRV (Melenovsky et al., 2003). There seems to be little consensus in the literature as to what the effects of SSRIs are on HRV (van Zyl et al., 2008; Kemp et al., 2010; Licht et al., 2010). However, a recent large independent cohort study showed that different antidepressants (e.g., tricyclic antidepressant, SSRI) were related to lower HRV (Kemp et al., 2014). The fact that the present recovery prediction model fails for the HRV data for the participants on heavy medication, again demonstrates the strong masking effect medication has on the HRV.

The participants in this study were adult male forensic inpatients. This is a group for which cognitive and executive functions deficits have been demonstrated before (Galski et al., 1990). Thus, based on the very high RMSSD during recovery in these participants on a mix of medication, one can speculate whether the level of HRV during recovery may add some information about the physiological effort invested in the task before the recovery. The results fit a previously observed pattern, where increased HRV was observed after exposure to a similar experimental mild-stress procedure (Hansen et al., 2003). Another study (Hansen and Johnsen, 2013) investigating the relationship between levels of neuroticism and performance on non-executive functioning tasks (tasks based on processes driven automatically), in both non-threatening and threatening situations, showed that participants with high neuroticism exposed to a threat had a significant increase in HRV from the task to recovery. This effect was absent when high neuroticism participants were not exposed to threats. For low neuroticism participants, this effect was absent regardless of threat level. In this latter study it was speculated whether the absence of threat in the recovery period caused the significant increase in HRV from test to recovery, since subjects with high neuroticism usually are characterized by low HRV. However, more research is needed to conclude whether information from the recovery measure can add something significant concerning mental and physical health.

Recently it was reported a strong relationship between tricyclic antidepressant and coronary heart disease (Kemp et al., 2015). Thus, as persons on heart medication and anti-depressants on average can be regarded as having poorer health (and thus lower life expectancy) than the general population, it is of interest to look deeper into the role of a fish diet for this group. The score and loading plots in **Figures 6  and 7** generate the hypothesis that the participants using heart medication and/or anti-depressants throughout the fish intervention period experienced a larger reduction in LF than the ones not using drugs. Thus, considering the debate about the autonomic origins of LF power (Thayer et al., 2010; Xhyheri et al., 2012), the fact that 2 min data epochs provide better estimates of LF (Thayer et al., 2010), and the present findings, one could speculate whether the LF represents the sympathetic activity, and that intake of fatty fish may be even more beneficial for the participants on heart medication. However, there is empirical evidence that LF power correlate highly with HF power (Thayer and Lane, 2007). Because of this and the small sample size, further investigation is necessary.

As with many diet intervention studies, the number of participants in this study is low. This becomes even more evident if trying to control for the different types of medication in use. The number of participants is not large enough to warrant a more thorough analysis of the effects of the different medicines. One may also speculate as to whether the effect of the medicines may really be due to the underlying pathology, and not the medication. There is a debate concerning this relationship and a recent study focusing on depression demonstrated that reduction in HRV was related to pathophysiological mechanisms rather than the effect of antidepressant (SSRI; see Brunoni et al., 2013). In the present study, all participants diagnosed with CVD or depression was on medication. To discern between medication and pathology is therefore not possible using this data set. Still, there are numerous reports in the literature about the effects of various medications on HR and HRV, and the effect we observe in our study thus seems reasonable. The consistency in which the participants group when controlling for medication gives credence to the findings presented here. The focus on the change data and the subsequent explorative multivariate analysis is not meant to replace traditional statistics. We have also employed *t*-tests on the latent variables to indicate whether the observed groupings are reasonable. Used in this way, this approach represents a valuable addition to the data analytical tools normally employed.

The original motivation behind this intervention experiment was to look at the effect of the nutrition on mental health (e.g., Hansen et al., 2014a,b). The *physical* health benefits of introducing fatty fish to our diet has been amply demonstrated in earlier publications. Van Horn et al. (2008) present a thorough review of this dietary aspect with regards to CVDs. For institutions having a clientele with a lower than average life expectancy, this effect should be factored in when designing health improvement plans. The question that remains to be answered is whether increased fatty fish consumption can enhance the effect of traditional medical treatment such as antidepressants and heart medication and further increase the life expectancy. It has been reported that while anti-depressants reduce the symptoms of depression, they may leave the HRV at sub-optimal levels. As HRV is a strong predictor of cardiac status, this indicates that persons suffering from depression are vulnerable to CVDs. Thus, based on the present data exploration it can be hypothesized that the beneficial effects of an increased fatty fish intake are even larger for this group. More knowledge about this will have important implications with regards to the development of health improvement interventions in psychiatric institutions.

## AUTHOR CONTRIBUTIONS

All authors have contributed to the design, analysis, interpretation, or acquisition. Drafting, revision, and final approval has been conducted by all authors. All authors agree to be accountable for all aspects of the work.

## Conflict of Interest Statement

The authors declare that the research was conducted in the absence of any commercial or financial relationships that could be construed as a potential conflict of interest.
